# Cost-effectiveness analysis of a state funded programme for control of severe asthma

**DOI:** 10.1186/1471-2458-7-82

**Published:** 2007-05-17

**Authors:** Rosana Franco, Andreia C Santos, Harrison F do Nascimento, Carolina Souza-Machado, Eduardo Ponte, Adelmir Souza-Machado, Sebastião Loureiro, Maurício L Barreto, Laura C Rodrigues, Alvaro A Cruz

**Affiliations:** 1 Programa para o Controle da Asma e da Rinite Alérgica na Bahia (ProAR) – Faculdade de Medicina, Universidade Federal da Bahia (UFBA), Salvador, Bahia, Brazil; 2 Instituto de Saúde Coletiva, (UFBA), Salvador, Bahia, Brazil; 3 London School of Hygiene and Tropical Medicine, University of London, UK

## Abstract

**Background:**

Asthma is one of the most common chronic diseases and a major economical burden to families and health systems. Whereas efficacy of current therapeutical options has been clearly established, cost-effectiveness analysis of public health interventions for asthma control are scarce.

**Methods:**

81 patients with severe asthma (12–75 years) joining a programme in a reference clinic providing free asthma medication were asked retrospectively about costs and events in the previous 12 months. During 12 months after joining the programme, information on direct and indirect costs, asthma control by lung function, symptoms and quality of life were collected. The information obtained was used to estimate cost-effectiveness of the intervention as compared to usual public health asthma management. Sensitivity analysis was conducted.

**Results:**

64 patients concluded the study. During the 12-months follow-up within the programme, patients had 5 fewer days of hospitalization and 68 fewer visits to emergency/non scheduled medical visits per year, on average. Asthma control scores improved by 50% and quality of life by 74%. The annual saving in public resources was US$387 per patient. Family annual income increased US$512, and family costs were reduced by US$733.

**Conclusion:**

A programme for control of severe asthma in a developing country can reduce morbidity, improve quality of life and save resources from the health system and patients families.

## Background

The prevalence of asthma is increasing in many countries [1,2]. This trend has been attributed to adoption of western lifestyle, and it is expected that prevalence of asthma will continue to increase in developing countries. The prevalence of uncontrolled severe asthma has also growing with great economic and social impact for families and health systems, major health resource utilization, loss of productivity and deterioration in quality of life.

The primary aim of persistent asthma management is to gain symptom control, by monitoring clinical manifestations of airway inflammation and regular use of inhaled corticosteroids [3,4]. But the proportion of individuals with persistent asthma reporting use of inhaled corticosteroids is low: 41% in Europe, 35% in the U.S.A., 14% in Asia and only 5% in Brazil [5,6].

Brazil has a high prevalence of asthma, with estimated 10 million people suffering from the disease [9]. In Salvador, a City located in the State of Bahia, the prevalence of asthma among adolescents estimated in the first International Study of Asthma and Allergy in Childhood (ISAAC) survey was 27,1%, one of the highest in the world [10]. In spite of this high prevalence, the standard strategy for asthma management in the public health system in Brazil is limited to treatment of exacerbation with bronchodilators and systemic corticosteroids. The combination of a high prevalence and the lack of access to secondary prevention through pharmacotherapy in the public health system, leads to high morbidity and unacceptable mortality [9]. In 2003, a pilot programme for management and control of severe asthma started in an outpatient clinic of Federal University of Bahia School of Medicine. The programme is offered by the state health system and includes specialized care, patient education and free inhaled medication for asthma and rhinitis [11].

Resources for public health actions are always limited, and therefore it is crucial for policy makers [8], to have evidence from clinical and economic studies, to define their priorities and to balance costs and effectiveness of available alternatives. This is true for both wealthy and developing countries. We hypothesized that severe asthma generate so high economic burden on the health care system and families that an intervention programme offering the best management for severe asthma would benefit patients without increasing costs. The study reported here was a cost-effectiveness analysis of this programme for management and control of severe asthma in Brazil, considering government and family direct and indirect costs involved in asthma management.

## Methods

### The Programme for control of asthma and Allergic Rhinitis in Bahia (ProAR)

This programme was organized by Federal University of Bahia Medical School and obtained operational support from the Unified Health Service (in Salvador, a unified public health system, comprising the Department of Health of the City of Salvador, the Department of Health of the State of Bahia and Brazilian Ministry of Health [11]). ProAR aims were to reduce symptoms and prevent exacerbations in severe asthmatics, with an integrated approach by a team of chest physicians, allergists, pediatricians, nurses, pharmacists, social workers and psychologists. Asthma education sessions are offered to patients and family members. Inhaled corticosteroids (budesonide) combined with long acting bronchodilators (formoterol) are given with no cost to all patients, as recommended by Global Initiative for Asthma (GINA) guidelines [12]. Nasal topical corticosteroids are also given to those with concomitant chronic rhinitis. ProAR started recruiting patients with severe asthma in Salvador in 2003; by October 2006, 1796 patients had been recruited into the programme.

### Participants

Eighty-one patients with severe asthma, from 12 to 75 years old, living in Salvador and its metropolitan area, were consecutively selected from those attending regularly the referral clinic for ProAR. To be included in the study, patients were required to have more than one year of severe asthma according to the classification by the Global Initiative for Asthma [12]. Therefore, the patients typically had continuous asthma symptoms, as well as daily limitation to exercise, frequent exacerbations and night symptoms, requiring daily use of a bronchodilator. The majority reported frequent emergency visits, hospitalizations, and some had been admitted to intensive care units. They also had a low forced expiratory volume in one second (FEV1) on spirometry. Additionally, patients were required to have no contraindications for the use of the inhaled corticosteroids and/or long acting beta 2 agonists bronchodilators, no concomitant lung disease (as assessed by history and chest X-Ray); to be non-smokers or have a smoking history < 10 pack/years and be capable of giving written informed consent. Recruitment was limited by convenience to patients admitted to the programme between April and September of 2004.

### Study design and evaluation procedures

This is a "before and after study" (pre-post study) of 81 patients with severe asthma, managed in ProAR. The objective was to compare the cost-effectiveness, for families and the public health system, of two strategies for severe asthma management of the same patients: the regular asthma care available in the Salvador public health system they used before joining ProAR and that offered by ProAR. The analysis considered the management of asthma one year before and one year after admission to ProAR.

**Figure 1:**
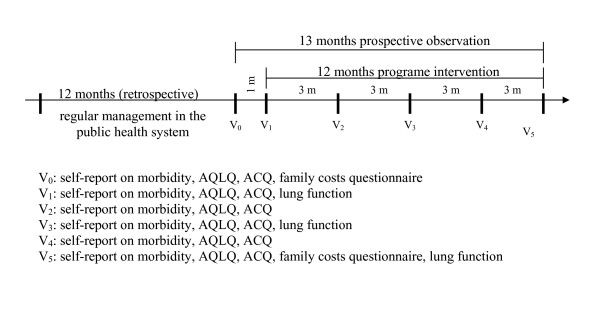
Study design and evaluations schedule.

Figure 1 summarizes the chronology of the study procedures. Upon referral to the programme, the selected patients were invited to participate in this study, to sign an informed consent form, and to answer the AQLQ (Asthma Quality of Life Questionnaire) and the ACQ (Asthma Control Questionnaire) that are standard instruments to measure asthma control and asthma-specific quality of life, both of which had previously undergone linguistic validation to Portuguese and are available electronically [13-16]. A month later they had their first medical consultation in ProAR, when they were seen by a specialist and received free medication. Both ACQ and AQLQ questionnaires were repeated during this visit. Afterwards, the ACQ and AQLQ questionnaires were answered every 3 months during the one-year of follow up. The two initial repeated questionnaire scores one month apart were taken as the patients' baseline status of asthma control and quality of life before intervention. During the initial visit researchers collected economic and clinical retrospective information regarding asthma treatment in the past 12 months, including patient's and family's income, expenses with transportation, doctor's visits, medications, therapeutical devices and diagnostic tests, emergency room visits, hospitalizations and intensive care admissions due to asthma. The same information was then collected prospectively on monthly visits during ProAR follow up and intervention for one year, for comparison with the previous year. Patients performed lung function tests to obtain forced vital capacity (FVC), forced expiratory volume in 1 second (FEV1) and peak expiratory flow rate (PEF) at baseline, before joining ProAR, and at 6 and 12 months of follow up to objectively evaluate their asthma control status [17]. The Ethics Committee of Human Research – School of Medicine of Federal University of Bahia, approved the study.

### The economic analysis

In order to compare the cost-effectiveness of treatment of severe asthma for the two strategies (regular public health system care and the ProAR), the costs for the public health system, for ProAR and for the families were estimated using accounting procedures. All these costs have been brought up to values at the time of the main analysis (February 2006) and the necessary depreciation estimated.

Costs were estimated for the public health system and for ProAR: health related direct costs for each patient, before and after their inclusion on the programme. The costs of outpatient care within the year before intervention, comprising doctor's visits, medications and diagnostic tests were calculated based on patient' retrospective information and using government official reimbursement values and pharmaceutical retail price. The costs of hospitalization, emergency room visits, and intensive care unit admissions, both after and before joining ProAR, were estimated based in Health Department of State of Bahia databases and public and charity hospitals' financial department surveys. To estimate the average cost of a day of hospitalization for a case of asthma we took into account the average total direct costs with hostelry (staff, current expenses, equipment and facilities depreciation, office and medical supplies), the number of total patients and the proportion of patients with asthma hospitalized per year, and the costs of specific asthma medication. The costs of ambulatory health care in ProAR were estimated as follows: the total annual stable costs with current regular expenses (administration, water supply and energy consumption, security, cleaning, maintenance), office and medical supplies, communications and staff, divided by the total number of patients admitted, to obtain the cost per patient/year. The variable costs were estimated by analyzing individual expenses with diagnosis and treatment.

Family costs**- **family's direct and indirect costs were estimated by using the Asthma Family's Costs Questionnaire (AFCQ), which collects information on the costs incurred by the families due to asthma treatment of a family member. This was adapted from a questionnaire previously created to estimate family costs of tuberculosis in Brazil [18]. The AFCQ reliability and reproducibility were evaluated and confirmed in asthma among 30 patients of our programme. The AFCQ is available electronically [19] and detailed results of its validation have been a matter of a paper that has been submitted to publication. This instrument has 33 questions divided into sets of items: family income, financial help, transportation expenses, loss of job, school absence due to asthma, medicines purchase, other expenses and loss of time waiting or moving to health services. The questionnaire was applied at baseline, to quantify expenses over the year before patient's admission to ProAR, and again at the end of study, to measure costs to the family during the year of ProAR intervention.

Cost-effectiveness analysis **- **the analysis was conducted to compare two treatment strategies for severe asthma. The first one is the regular treatment offered by the public health system in Salvador, in which patients received treatment only to control exacerbations with no regular inhaled or oral preventive medications or regular follow up. The second approach, in the same patients, after admission to ProAR and being followed prospectively for one year receiving multiprofessional regular care, free inhaled preventive medication and educational sessions. The effectiveness of the intervention was measured as "hospitalizations avoided" by the programme. Cost-effectiveness incremental analysis was performed by comparing the costs and health results, dividing the difference in costs per the difference of health results obtained on each strategy [20].

Sensitivity analysis – this was carried out to ascertain the reliability of the cost-effectiveness analysis under varied assumptions regarding costs of health care. We challenged our main findings by repeating the cost-effectiveness analysis while changing the estimated hospitalization cost from our real life calculus to what was reimbursed to the State or City Departments from the Ministry of Health, which was 76% less [21].

### Statistical analysis

The sample size was calculated by Stats Direct 7.0 software considering the estimated ProAR intervention effect of reducing hospitalizations during one year, as compared to the year before. Hospitalizations are an important parameter to assess asthma control, morbidity and costs [12]. The minimum sample size required to detect as statistically significant a 50% reduction in costs per patient/year was 58 patients, for a power of 80%. We increased the sample to 81 patients in order to compensate for probable losses of follow up or mortality of patients with such severe disease.

Data were entered onto a Microsoft Excel^® ^and SPSS 11.5 spreadsheet for statistical analysis. Two tailed tests were carried out and p values lower than 0.05 considered statistically significant. The categorical variables were reported as proportions and compared with Chi-square Tests. All continuous variables were compared by using Wilcoxon Signed Rank Test [22].

## Results

### General characteristics of patients

Most patients came from a poor socio economic stratum, having a median family income of US$ 2,768 per year. Many worked in the informal labor market and 14 (17%) were unemployed. They had low educational level: 68% had less than 5 years of schooling. The majority of patients were of mixed ethnicity, predominantly African origin and 68 (83%) were female. This is consistent with the literature [22]. They had asthma for an average of 26 years and 22% had another associated chronic disease. The mean age of patients was 45 ± 16 years, and we found that severe disabling asthma had a major impact in their economical activities and indirect costs to their family. Forty one percent of the families reported loss of a job by patients or their parents, 5% of the patients had retired early and 6% never worked because of the asthma (Table 1).

**Table 1:** Demographic, clinical and socioeconomic characteristics of the patients included (N = 81)

**Age (average +/- SD)**	45 ± 16
**Ethnicity (n/%)**	
Caucasian	10 (14)
African descendents	64 (86)
**Female gender (n/%)**	68 (84)
**Occupation (n/%)**	
Employed	29 (35)
Unemployed	14 (17)
Housewife	15 (19)
Retired	12 (15)
Student	11 (14)
**City of residence (n/%)**	
Salvador	76 (94)
Surrounding cities	5 (6)
**Education (n/%)**	
Illiterate	14 (18)
Middle School completed	40 (50)
High School completed	22 (27)
University completed	4 (5)
**Private health insurance holders (n/%)**	13 (16)
**Presence of comorbities (n/%)**	17 (22)
**Duration of diagnosis of asthma in years (average +/- SD)**	26 ± 17
**Asthma's restrictions of the patient's or families activity (n/%)**	
School absence	9 (11)
Never worked due to asthma	5 (6)
Early retirement due to asthma	4 (5)
Loss of job by the patient or family	33 (41)
**Family annual income (US$) (median/quartiles)**	2768 (1912/4033)

Data from the retrospective phase

Of the 81 patients included, 64 (79%) concluded the study. In spite of the regular treatment offered, 3 (4%) patients died during the one year follow up. A total of fourteen (17%) patients did not complete the study. These patients had different characteristics from the compliers: they were younger, of a better socioeconomic status, had less comorbidity, and better access to private health care. Some noncompliers reported having left the programme because of the time spent in the regular visits answering our questionnaires.

### Health aspects

When comparing reported data for the year before registration in ProAR to the year after joining for the 64 patients that concluded the follow up, we found an increase in the use of inhaled corticosteroids, in scheduled specialist visits and spirometries performed and a substantial reduction in hospitalizations, in intensive care unit admissions and in emergency room visits/unscheduled consultations. Lung function improved 47% in PEF and 10% in FEV_1_. ACQ scores improved 29% and quality of life, as measured by mean overall AQLQ, improved 29% (Table 2). In the one-month baseline period (between V_0 _and V_1_, the two visits performed previous to intervention in ProAR), the AQLQ and ACQ scores remained the same. After 3 months of medication, the scores reached significant improvement, and that was maintained until the end of the one-year follow up period.

**Table 2:** Comparison among two strategies for severe asthma treatment concerning health care utilization, quality of life and asthma control (n = 64)

	year before ProAR	year after ProAR	
**Health care utilization per patient**	median (quartiles)	median (quartiles)	p*
Regular specialist visits	0 (0/0)	9 (6/12)	< 0.01
Spirometries performed	1 (0/1)	2 (2/2)	< 0.01
Emergency/unscheduled visits	36 (6/120)	1 (0/3)	< 0.01
Hospitalizations	1 (0/2)	0 (0/0)	< 0.01
**Quality of life**			
Total AQLQ score	2 (2/3)	4 (3/6)	< 0.01
**Asthma control**			
ACQ scores	4 (3/5)	2 (0.6/3)	< 0.01
% of expected FEV_1_	69 (49/82)	76 (58/84)	0.560
% of expected PEF	45 (34/66)	66 (49/83)	< 0.01

* Wilcoxon Signed Rank Test

The average duration of interviews was 30 minutes for AFCQ, 20 minutes for AQLQ and 10 minutes for ACQ. Nine percent of patients did not perform spirometries because of functional limitations and 5% did not answer the AQLQ because of insufficient intellectual ability.

### Economic issues

Table 3 shows that, when the 64 patients have controlled their asthma in ProAR, the increase in preventive treatment costs including regular inhaled medication, lung function tests, costs of educational and multidisciplinary staff was largely compensated by a reduction in hospital costs. This has lead to a total annual economy of US$ 387 to the public health system per patient, and therefore savings of US$ 24,768 in public health resources for the cohort of 64 patients. There was also savings for the family: the family's annual income increased 18% (US$ 512/family/year) and the total costs for the families, comprising expenses and loss of income, were reduced by 90% (US$ 733). The proportion of total family income spent with asthma treatment decreased from 29% to 2%. This resulted in a median annual economy of US$ 1,245 per family, when we consider the increase in income and the reduction of total costs. There was a reduction of median time spent with transportation per patient, from 6 hours to three hours/month (p < 0.01), and in time waiting on health services due to asthma from 4 hours to 1.7 hours/month (p = 0.05).

**Table 3:** Comparison among economic parameters of two strategies for control of severe asthma (n = 64)

	year before ProAR (US Dollars)	year after ProAR (US Dollars)	
	median (quartile)	median (quartile)	p*
**Government annual costs of treatment per patient**			
Cost of outpatient treatment	184 (177/192)	359 (240/471)	< 0.01
Cost of hospital treatment	590 (78/1330)	0 (0/19)	< 0.01
Total annual government costs of treatment	750 (286/1462)	363 (255/481)	< 0.01
**Family annual costs of treatment and income**			
Family income	2768 (1912/4033)	3280 (2191/5050)	0.02
Family expenses with asthma (direct cost)	615 (343/1374)	74 (34/275)	< 0.01
Proportion of income spent with asthma	29%	2%	
Losses for patient and companion (indirect cost)	0 (0/134)	0 (0/0)	< 0.01
Total family costs (direct + indirect)	807 (358/1509)	74 (35/281)	< 0.01

* Wilcoxon Signed Rank Tests

**Figure 2:**
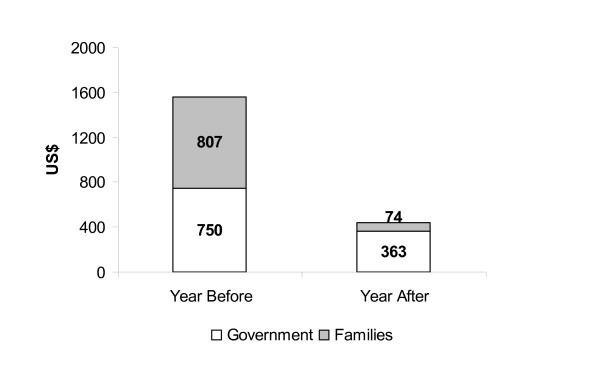
Burden of disease: severe asthma annual median costs per patient, and proportion paid by government and families, before and after ProAR intervention.

The evaluation of the burden of asthma in this sample of patients shows that when patients were treated in the usual public health system low socioeconomic strata families paid for 52% of the costs of severe asthma while the government paid 48%. This contrasts with patients registered in ProAR, with the poor families paid for only 17% of the costs of the management of severe asthma (Figure 2).

Incremental cost-effectiveness analyses demonstrated that, for each hospitalization avoided there was a saving of US$ 1,395 for families and government together. The incremental cost-effectiveness analysis had a negative result, which means that ProAR strategy saves resources and decreases morbidity (Table 4).

**Table 4:** Cost-effectiveness incremental analysis of the strategies for treatment of 64 severe asthma patients

**Strategies**	Costs (US$)	Hospitalizations	Health Result	Incremental Cost (US$)	Cost-effectiveness incremental
			(C-D)	(B-A)	(F/E)
**Intervention 1**					
The usual treatment of severe asthma offered by the public health system with medication for exacerbations	**173,440** **(A)**	**85** **(C)**			
					
**Intervention 2**					
Treatment of severe asthma offered by ProAR with inhaled corticosteroid + long acting bronchodilator	**56,256** **(B)**	**1** **(D)**	**84** **(E)**	**-117,184** **(F)**	**-1,395**

Main result: there was an economy of US$ 1,395 per hospitalization avoided.

The effectiveness of the intervention was measured as "hospitalization avoided" by the programme and the costs are including the families and governments costs.

The sensitivity analysis we have performed by changing the cost of hospitalizations has demonstrated that, even considering only the fraction of the public hospitals costs reimbursed by the federal government, ProAR strategy of treatment remains more cost-effective than the usual public health system (Table 5).

**Table 5:** Sensitivity analysis of the strategies for treatment of 64 severe asthma patients

**Strategies**	Costs (US$)	Hospitalizations	Health Result	Incremental Cost (US$)	Cost-effectiveness incremental
			(C-D)	(B-A)	(F/E)
**Intervention 1**					
The usual treatment of severe asthma offered by the public health system with medication for exacerbations	**133,991** **(A)**	**85** **(C)**			
					
**Intervention 2**					
Treatment of severe asthma offered by ProAR with inhaled corticosteroid + long acting bronchodilators	**56,256** **(B)**	**1** **(D)**	**84** **(E)**	**-77,735** **(F)**	**-925**

Main result: there was an economy of US$ 925 per hospitalization avoided.

The effectiveness of the intervention was measured as "hospitalization avoided" by the programme and the costs are including the families and governments costs.

## Discussion

This study demonstrated that an intervention programme that provides regular specialized care to severe asthmatics, including free pharmaceutical assistance, is more effective in controlling severe asthma and improving quality of life, at a much lower cost, than the regular treatment usually provided in the public health system, in a sample of underserved population in Salvador – Brazil. The clinic and economic impact of the strategy of ProAR in treatment of severe asthma was so high for families and public health system, that a small sample size of patients was sufficient to demonstrate a statistical significant advantage in favor of the programme.

Salvador has 2,614,840 inhabitants, and 67% of them use the public health system [24,25]. The results of this study, providing evidence that the new intervention has a lower cost and greater effectiveness than the regular care, is of major public health importance to other low and middle income countries [19] and should lead to implementation of policies that will benefit a large number of patients [26]. The finding that organized specific programmes for asthma control can be more cost-effective is new for developing countries, but this has been shown before for developed countries: the National Finnish Programme for Asthma Control for example, has shown that, after 10 years of implementation, it reached significant reduction in direct costs with hospitalizations, deaths, disability and early retirement, despite continued growth of asthma's prevalence [27].

Incremental cost-effectiveness estimates, in which concomitant cost and benefit advantages are detected deserve careful interpretation. Taking this into consideration our sensitivity analysis was as conservative as possible. Although we are confident that our results and conclusions are accurate for this setting, more research is needed on the impact of programmes for control of severe asthma in other populations of severe asthmatics from low to middle income countries. Information on costs of severe asthma to families is scarce [3,27], although many studies of cost to the public health system have been reported [28]. The present study provides a better estimate of the complete economic impact of severe asthma, by including family and public health service costs. The AFCQ, used for the first time in this study, was introduced as an easy and useful tool to estimate family costs. It has shown that the economic burden of severe asthma was shared equally by low-income families and government before ProAR. This suggests that programmes like ProAR will also have major socio-economic benefit to families of severe asthmatic patients.

An Australian study estimated the annual family's costs for treatment of 238 asthmatic children. It found that the mean annual treatment cost per asthmatic children was US$ 164 [29]. In the present study, the annual family costs (US$ 807) are five times higher. Possibly, this difference is due to the severity of the asthma of our patients and because we took into consideration indirect costs too. In California (USA), a study that analyzed annual costs of asthma according to the severity of disease, found that costs of severe athma was US$12,813 per patient/year [3]. In the present study, the total annual burden of disease per severe asthmatic patient, including family and government cost, was of US$ 1,557; eight times less than the Californian study. This may reflect a smaller investment in asthma control and/or reduced costs of treatment in Brazil, when compared with a developed country.

The success of asthma treatment in preventing exacerbations seems to be associated with regular adequate measures. Patients with scores of ACQ under 1.5 are considered under control [16] (This is used as the cut off point for clinical trials). Within one year of follow up, patients treated in ProAR, with asthma of 25 years duration on average, reduced the ACQ score from four (poorly controlled) to two, which is very close to adequate control. Lung function measurements (FEV_1 _and peak flow) were less responsive to changes in asthma control than ACQ scores, in this study, which is in agreement with previous reports.

Severe or uncontrolled asthma causes limitations and has an impact in physical, social, and emotional well-being of patients and their families. Its control may result in remarkable change in quality of life [15]. After ProAR intervention, patients left a situation of extreme limitation (AQLQ mean score of 2) to enter a mild/moderate limitation zone (AQLQ mean score of 4) during the one year of intervention.

The main limitation of our study is that information from the year before intervention was collected retrospectively from patient reports. It would not be ethical to have a parallel control group of severe asthmatics followed up without access to ProAR, once free preventive inhaled medication was made available. Therefore, the only way we could study patients inside and outside the programme was comparing their own profile before and after the intervention. In this study, patients apparently had no difficulty to recall hospitalizations, emergency room visits, income, financial help, medicine prices, and transportation expenses. They were able to document their recent expenses with medicine bringing in boxes and/or drugstore receipts and some hospitalizations and emergency visits with medical reports and prescriptions. The presence of an interviewer might have influenced the patient's answers. However, this was needed as the majority of patients had low educational level and some were illiterate. The interviewer was trained to avoid influencing the answers, and was the same for all patients during all the study [30].

## Conclusion

In conclusion, this study has demonstrated that, in a developing country, a public health programme aimed to control severe asthma offering specialized multidisciplinary care in a reference clinic, based on secondary prevention of symptoms and exacerbations with a combination of inhaled corticosteroid and long acting beta 2 agonist bronchodilator (given for free), and patient's education, is a more cost-effective alternative to manage severe asthma than the previous regular public health approach of treating exacerbations only. The reduction in government and family direct and indirect costs is so remarkable that it compensates all the costs involved in running the programme and supplying effective medicines, with incremental economy to the government and to families' budgets. And above that it has improved asthma control and asthma related quality of life. A well-structured programme for asthma control, focused in severe cases, can reduce morbidity, improve quality of life, and decrease health related costs in middle income countries.

## Competing interests

The author(s) declare that they have no competing interests.

## Authors' contributions

RF, AAC, MLB, and LCR designed the study, supervised field work, analyzed data, interpreted results, and wrote the paper; ACS designed the study and contributed to the analysis; HFN, CSM, EP and ASM did field work, interpreted results, and edited the paper; SL interpreted results and edited the paper.

## Pre-publication history

The pre-publication history for this paper can be accessed here:


